# “It will always continue unless we can change something”: consequences of intimate partner violence for indigenous women, children, and families

**DOI:** 10.3402/ejpt.v5.24585

**Published:** 2014-09-12

**Authors:** Catherine E. Burnette, Clare Cannon

**Affiliations:** 1 School of Social Work, Tulane University, New Orleans, LA, USA; 2 City Culture +Community, Tulane University, New Orleans, LA, USA

**Keywords:** American Indian, Native American, domestic violence, family violence, qualitative, ethnography, life history

## Abstract

**Background:**

Violence against indigenous women and girls is endemic, yet the absence of research on the consequences of this violence from the perspectives of women presents a profound barrier to the development of knowledge, along with violence prevention and mitigation. Although family is central to many indigenous communities, existing research typically examines the consequences of intimate partner violence (IPV) on women or children in isolation, rather than examining its consequences holistically.

**Objective:**

The purpose of this article is to identify US indigenous women's perspectives about the impact of IPV on women, children, and families.

**Method:**

Data were collected with 29 indigenous women affected by violence from a Southeastern tribe in the United States. As part of a larger critical ethnography, pragmatic horizon analysis of life history interviews revealed the consequences of IPV across multiple levels.

**Results:**

Women reported profound psychological consequences resulting from IPV. The majority of women had witnessed IPV in their childhood, providing support for an intergenerational cycle of violence. Women reported psychological consequences on children, which paralleled those reported by women, leaving deep impressions on children across their life course. Consequences on children and whole families were extensive, indicating the negative ramifications of IPV transcended personal boundaries and affected children and families across multiple generations.

**Conclusions:**

Given the tight-knit nature of indigenous families and communities, the consequences across individuals and families were noteworthy. However, a dearth in research examining consequences of IPV across levels fails to capture the interconnections of consequences for women, children, and families. Given the centrality of family in many indigenous communities, examining IPV from a holistic perspective that incorporates multiple levels is recommended for IPV research and intervention development.

As stated by the United Nations (2013), “Violence against indigenous women and girls is endemic in every part of the world” (p. 22) and this violence has been linked to the *historical oppression* (Burnette, 2014), or the intergenerational experiences of subjugation imposed upon indigenous populations throughout colonization and into the present. Regardless of whether nations have significant populations of indigenous populations, all countries have been affected by colonization; with nations’ associations with colonization comes a concomitant responsibility to understand its insidious consequences. Thus, the ethical responsibility for researchers and clinicians to understand indigenous women's experiences of traumatic violence transcends national and international contexts. For this article, we define *indigenous* people as those who have been affected by colonization and are thought to be original inhabitants of a land, in this case the United States.

Violence against indigenous women has drawn national and international attention. However, the absence of research on the experiences of indigenous women and girls specifically, presents a profound barrier to knowledge development and violence prevention and mitigation (United Nations, 2013). Intimate partner violence (IPV) is one of the most common forms of violence against indigenous women and it includes the physical, psychological, or sexual harm caused by a current or former partner (Centers for Disease Control and Prevention, 2013). IPV is a widespread social problem in the United States (US), yet indigenous women experience it acutely, at a rate 2.5 times the rate of all non-indigenous women (Greenfield & Perry, 2004; Tjaden & Thoennes, 2000). According to Black et al. (2011), 46% of indigenous women experience IPV, which is more likely to result in injuries and require medical care than IPV among the general population (Bachman, Zaykowski, Poteyeva, & Lanier, 2008). Despite these rates, great variability exists across the 566 federally recognized (Indian Health Service, 2014) and approximately 400 non-federally recognized tribes (U.S. Government Accountability Office, 2012). Indeed, rates for women in population based indigenous communities ranged from 46 to 91%, whereas the rate for non-indigenous women ranged from 7 to 51% (Oetzel & Duran, 2004). Thus, although variability is evident, the rates of IPV for indigenous women tend to be disproportionately high.

## Consequences of IPV on indigenous women, children, and families

Given the centrality of tight-knit families in many indigenous communities (Burnette, 2014), the ramifications of IPV extend beyond individual partners. Research has found IPV impacts the mental health of affected partners (Coker et al., 2002), children (MacDonell, 2012), and families (Sturge-Apple, Skibo, & Davies, 2012). Despite epidemic rates and its effects across multiple levels, there is a dearth of research on the impact of IPV on indigenous women, children, and families (Bohn, 2003; Oetzel & Duran, 2004), critical information about the interconnections among the consequences of IPV across women, children, and family may be missed. This absence poses a critical gap in knowledge and is a barrier to IPV prevention and amelioration. Therefore, the purpose of this article is to identify indigenous women's perspectives on the impact of IPV on women, children, and families, along with the interconnections among these consequences.

Despite the paucity of research on IPV among indigenous women in the United States (Bohn, 2003; Oetzel & Duran, 2004), research on the consequences of IPV is even more limited. Only five empirical studies were found on the consequences of IPV among indigenous women in the US (Bletzer & Koss, 2006; Bohn, 2003; Evans-Campbell, Lindhorst, Huang, & Walters, 2006; Norton & Manson, 1995; Oetzel & Duran, 2004), and no studies were found on the consequences of IPV on children or families. Researchers have found that violence against indigenous women tended to begin at a young age and was related to depression, substance abuse, and attempted suicide (Bohn, 2003). Norton & Manson (1995) reported women feeling more stressed and depressed, and 38% felt their substance use worsened since experiencing IPV. Moreover, Bletzer and Koss (2006) described the Cheyenne subsample emphasizing the lifetime impact of rape experiences. Although Evans-Campbell et al. (2006) reported IPV being associated with a high degree of emotional trauma, as well as depression and dysphoria, Robin, Chester, and Rasmussen (1998) found indigenous women reported rape victimization being significantly associated with psychiatric disorders, whereas IPV as a whole was not. The aforementioned research makes it apparent that research on the consequences of IPV for indigenous women is scarce, which is a fundamental problem, given its disproportionately high rates. Moreover, available research documents the variability of the consequences of IPV across indigenous communities, indicating the need for localized and context-specific examinations.

## Consequences of IPV on women, children, and families

The psychological consequences of IPV are significant. Victims of IPV are more likely to report depressive symptoms, substance abuse, and chronic mental illness than non-victims (Coker et al., 2002). Research documents IPV placing people at risk for anxiety, depression, posttraumatic stress disorder (PTSD), antisocial behavior, suicidal thoughts, and emotional detachment (Black et al., 2011; Coker et al., 2002; Stampfel, Chapman, & Alvarez, 2010).

Another major impact of IPV is their attendant negative effects on children (Graham-Bermann & Perkins, 2010). Children from households with IPV were five to seven times more likely to suffer psychological problems, such as childhood depression, anxiety, aggression, insecure attachment, and low self-esteem, than children from non-violent households (Cummings & Davies, 2010; Sturge-Apple et al., 2012). Consequences of witnessing IPV have been found to be moderated by gender, and Moretti, Obsuth, Odgers, and Reebye (2006) reported 46% of the girls and 22% of the boys in the study exhibited symptoms of PTSD. IPV has been found to increase risk for delinquency and violent expressions as well as impaired relationships with family, friends, and partners (MacDonell, 2012). Exposure to IPV places children at risk for challenges later in life, such as experience IPV as adults, psychiatric distress, self-harm, trauma symptoms, depression, underemployment, poorer educational attainment, and problems with attachment (Rossman, 2001).

In addition to negative consequences from IPV for partners and children, Sturge-Apple et al. (2012) reported that families are directly affected by IPV (psychological consequences related to exposure to violence) and indirectly affected by IPV (interparental conflict impairing parenting and parent–child relationships, which in turn constrains child development). Despite the recommendation of Sturge-Apple et al. (2012) for research that examines the consequences of IPV on whole families, no such research was found. Given the primacy of families within indigenous communities (Burnette, 2014), more research on how IPV affects whole families is clearly needed. IPV not only affects women, children, and families at one point in time, it has been found to be transmitted intergenerationally. Social learning theory proposes that violence can become normative for children who witness IPV who may, in turn, resort to the use of IPV as a form of conflict resolution (Cannon, Bonomi, Anderson, & Rivara, 2009; Whitfield, Anda, Dube, & Felitti, 2003).

The literature on effects of IPV on women, children, and families is predominantly quantitative studies, which produce a broad understanding; however, qualitative studies are necessary to understand the particular complexities of IPV and its consequences across multiple dimensions. Given the aforementioned gender differences (Moretti et al., 2006), examining experiences of women separately is warranted. With an absence of research on indigenous women and the absence in the broader literature that incorporates family, this article provides a holistic understanding about the reported consequences of IPV on women's lives across their multiple roles as partners, children, and family members. The purpose of this study is to identify indigenous women's perspectives on the consequences of IPV for women, children, and families.

## Methods

### Research design

As part of a larger critical ethnography (Burnette, 2014), data for this article is based on life histories collected with 29 indigenous women affected by violence from a Southeastern tribe in the United States. Critical ethnographies enhance understanding and provide explanations about social problems, such as the consequences of IPV on women, children, and families (Carspecken, 1996). Carspecken's (1996) method was chosen due to its comprehensive standards of rigor. It is recommended to explain social behavior in a holistic and systematic way (Hardcastle, Usher, & Holmes, 2006). Data were collected by the first author during the summer of 2012 while living adjacent to indigenous reservation communities of women. Carspecken's (1996) methodology includes five stages which have been followed in a culturally sensitive way and are described in detail in Burnette, Sanders, Butcher, & Rand, 2014) research. Moreover, the extensive efforts to engage in reflexive, culturally sensitive, and ethical research are described in other research (Burnette, 2014; Burnette, Sanders, Butcher, & Salois, 2011; Burnette, Sanders, Butcher, & Rand, 2014; Burnette & Figley, in press). For instance, precautions were taken, such as excluding any women who identified as being at a safety risk, having a licensed master's level social worker with extensive experience in crisis social work and suicide assessment conducting the interviews, and providing all women a resource lists of additional services.

### Sampling and setting

The centrality of family in indigenous communities tends to be paramount (Burnette & Sanders, 2014), and because women experience violence directly (as children and adults) and indirectly (through family members), indigenous female adults who had directly and indirectly experienced violence were purposively selected. Despite inclusion criteria, all participants experienced violence at some point across their lives. Recruitment efforts included community agencies and snow-ball sampling. Data reached saturation at 25 interviews, and four more interviews were collected for a total of 29 interviews. The sample sizes of other research using this methodology ranged from 8 to 23 (Carspecken, 1996; Dove, 2010; Mills, 2007). Women resided in multiple rural indigenous communities of a tribe in the Southeastern United States.

### Data collection and analysis

Life history interviews followed a semi-structured interview guide to answer the research question: What are indigenous women's perceptions of the consequences of IPV on women, children, and families? We define families to include *extended families*, which can include family members related by blood, along with clan, tribe, and adopted family relationships (Waller, Okamoto, Miles, & Hurdle, 2003). Examples of interview questions included, “If your parents were together during your childhood, describe for me what you remember about their relationship” and “Describe for me how you have been affected by IPV.” In-person interviews lasted approximately 2 hours, on average. Women were offered a copy of their professionally transcribed interview and received a $20 gift card for participation.

Data were analyzed using the method outlined by Carspecken (1996) *pragmatic horizon analysis*, which enables explicit and implicit meaning of data to be uncovered (For a comprehensive description of this analysis as applied to this research, see Burnette, 2014). The qualitative data analysis software program, NVivo was used. All themes reported in results were present among at least 50% participant's interviews. Three colleagues reviewed coding to uphold fidelity. Approximately 60% of participants engaged in member-checks and validated all themes with no changes made. Peer-debriefing was completed daily during data collection, and two cultural readers from the tribe reviewed and validated results. For an in-depth discussion of how all validity requirements were upheld, see Burnette, (2014).

## Results

Participants, aged 22–74, all had a high school diploma, and 33% held a bachelor's degree or above (see Burnette, 2014 for a table of demographics). With over 70% of women witnessing IPV as children, and often as adults, women tended to be affected by IPV along multiple dimensions. Thus, women not only recounted personal experiences of IPV, they recalled being exposed to IPV as children, as adult family members, and as friends. Due to the tight-knit nature of these indigenous communities, multigenerational households and multi-family households were common. Therefore, even if women did not personally experience IPV for a time, they tended to be exposed to it through their close connections to friends and family. The following results draw from women's personal experiences of IPV as well as their recollections of their exposure to IPV as family members.

### Consequences of IPV on women

Among participants, 65% of women spoke about personal consequences of IPV. Women tended to report severe and often life-threatening forms of abuse. One woman spoke about family members’ experiences with IPV:

She went through getting hit on the back of the head with a crowbar. She lost all her top teeth, as a result, for one domestic issue. I think he even attacked her with a knife and caused some damage to her hands. She is in very poor health now.

Indeed, women's experiences of IPV often resulted in trips to the hospital for medical treatment. A woman recalled, “He started hitting me, and I remember him knocking me down, and I guess he kicked me on the back of my head; and next thing I know, I was in hospital.”

Women reported psychological consequences, including PTSD, depression, and suicidal symptoms. Suicidal thoughts were frequently reported as consequences of IPV. One woman stated, “I was so tired that I even contemplated on suicide.” A woman recalled the aftermath of IPV for her family member, stating, “Twice she had attempted suicide. … she tried to overdose some medicine, some pills.” Other women reported significant depression. As a woman recalled, “I went through some depression. … I tried to go back to work, I just couldn't—I couldn't function.” One woman recalled the severity of her mother's depression after experiencing chronic IPV, “She holds on to it and it—it destroys her.” She added, “She has been admitted into a mental hospital like about three, four times. She has been through electric shock therapy.”

Posttraumatic stress symptomatology was frequently reported by women who experienced terror and fear of the perpetrator, even after the relationships ended. As a woman remembered, “It's horrible to go through that abuse, to be fearful all the time. What's going to happen next? What's going to happen when I leave home?” As another woman explained, “Psychologically he was in my head like all the time; every time I heard a noise, I thought he was coming to get me. I had nightmares that he was coming to get me.” This could lead to insomnia, as she added,

I couldn't get enough sleep because I was always having nightmares that he was coming to get me. And whenever I heard a noise around the house I kept thinking, you know, “He is here, and he has found me.”

As indicated, the consequences of IPV often persisted beyond the boundaries of the violent relationships, and women reported the ways it affected their relationships with others. A woman commented on her inability to trust after experiencing life-threatening IPV, stating, “I try not to get close to anybody as far as having feelings and trusting them.” Other women spoke about difficulty making decisions on their own. One woman stated, after her partner had controlled every aspect of her life, “Even in terms of small decisions like … what brand of toilet paper do you use?” She remembered,

It was always something wrong with my decision or my choice, and of course, he [perpetrator] always had a better choice … Now I find myself [when her current partner asks], “Where do you want to go out” [she replies] “It doesn't matter.”

This was a change from describing herself as “sure of myself” prior to the violent relationship. Another change she noted, “I would have accepted responsibility for everything [in the relationship] and … minimize his responsibility in the relationship, whereas I was not like that before.” By these results, it appeared that some women became conditioned to survive in the abusive situation by accommodating the partner, and this patterning affected their’ subsequent relationships.

### Consequences of IPV on children who witnessed IPV

This psychological conditioning aspect was apparent in the ways children were affected by exposure to IPV, and 64% of women spoke about consequences of this exposure. As one woman explained,

We have children who witnessed neglect, who witnessed abuse, who witnessed IPV in their home. And it will always continue unless we can change something. And just like the boys, you hear them make the remarks, “I am never going to do what Daddy does to Mom,” but they do; because they have been conditioned that, this is acceptable. I mean, even though they may not accept it in their mind, it's a way of life.

Many of women's earliest memories were of traumatic IPV. As a woman recalled, “The first memory I really had is of him [father] beating her [mother].” She added, “He beat her and we hid under the bed.” Another woman elaborated on the intergenerational cycle of witnessing IPV and experiencing it as an adult:

I actually saw him [father] take a pistol and hit her on side of the head, which caused her to go to the hospital … so it [the IPV I witnessed as a child] kind of applied to my marriage. Even though she was in the hospital with the injuries she had, he went to hospital and tried to finish her off over there, they had to call the cops.

Some children were caught in the crossfire of IPV, as explained, “I remember my sister yelling at my dad and telling him that it's his fault for going and screwing another woman, and because of that, he started yelling and trying to hit her.”

Women spoke about the consequences of children witnessing IPV. A woman recalled traumatic symptomology and felt frozen when witnessing IPV. She recalled,

He [father/perpetrator] is holding her [mother] down … and he is literally this [motions] close to her face and he is screaming, screaming, and screaming. … I'm just kind of standing there watching … so when she is coming through the door, he shuts the door to where literally half of her is in the room … and he is pushing it and pushing it, and pushing it.

Numerous times, this woman mentions watching in a frozen state in relationship to the trauma, as she described, “It was pretty much violence every day. Whenever they are fighting I kind of go back to that place where I'm just watching.”

One woman remarked on the psychological consequences of her children witnessing the IPV she experienced. Her children reportedly identified with the perpetrator after the relationship ended, as she explained, “They [children] were still telling my ex, where I was going, where I was staying, who I was seeing.” Likewise, a woman spoke of how a family member's children taunted their mother as her perpetrator did. This woman had made several attempts at suicide and her child would call her names, saying, “Shut-up, ‘Suicidal’.” Still another of her children demonstrated profound suicidal and aggressive behaviors, as she stated, “He tied up somebody else and had a knife on him.” This adolescent may have been reenacting the IPV he witnessed throughout his upbringing or dealing with his emotions in the way that was modeled to him.

Some women spoke of mental health consequences of their exposure to IPV as children including “bipolar” disorder, “depression,” “insomnia”, and “adult ADHD [attention deficit hyperactivity disorder].” One women stated, “I have actually been in the hospital four or five times … I was very suicidal.” Although her children provide her purpose to maintain focus in her life, suicidal thoughts have recurred, as she stated, “I think about death a lot. … I have like no desire to live.”

Children were not only directly affected by witnessing IPV, but were also indirectly affected by the concomitant unmet need for love and nurturance. One woman described her child, as stated,

I think she is the one that wanted a father the most and it hurt her the most. The way he treated her, the way he wasn't there for her when she would beg him to take her fishing … he was “too busy” to do that.

This child resorted to self-harm to cope with her pain. Another woman who witnessed IPV as a child described resultant depression and how she, “turned into a cutter for a while, I used to cut until probably I was 17.” She tried to fill her need for a nurturing father by picking partners that resembled him and, “looking for that love, that acceptance.” She remembered thinking, “He has got to love me somewhere deep down inside.” These attempts to recreate an experience where she felt the love she desired were relevant to her re-victimization, as she reported experiencing child sexual abuse and rape.

### Consequences of IPV on family

Often women recounted families and friends being significantly affected by their loved ones’ experiences of IPV, and 52% of women spoke of this. IPV tended to affect multiple family members intergenerationally. One woman spoke about IPV affecting not only her mother but her own life and the lives of her siblings. She recalled, “My brothers, they've done the same thing to their girlfriends or their wife. … My husband at one point had done the same thing to me.” Still another woman recalled how this violence was normalized, “My brother, he was very, very abusive to his wife, I mean, where he would literally pull her by the hair, and to him, it seems like it was okay.” Another woman understood the tolerance of IPV to be due, in part, to many family members experiencing it and it being normalized within the family. She stated, “With my family, especially with my grandmother and my aunt there. To them it was very acceptable for it to happen, they would never be angry at him for doing anything that he did.” She explained, “His sister was beat by her husband, my grandmother was beat by my grandfather, so to them it was very acceptable.”

Because many families were multi-family households, at least temporarily, if one family was affected by IPV, all members of the household were affected. One woman recalled witnessing her family member's experiences of IPV, remembering,

I spent some time at their house. I remember one morning, I just heard him [perpetrator] saying, “Get back in here”. … And just like that, he snapped and just started beating up on her right there.

Multiple participants had been affected by loved ones being killed through IPV. The profound effects of these deaths transcended the boundaries of blood-relation family members and into adopted friends as family members (Waller et al., 2003). As a woman stated, “My best friend was murdered from a IPV.” She added, “He [perpetrator] had beat her with everything he possibly could. … People were there—[they] didn't even try to stop it.” One family member of a woman killed through IPV described how her son had nightmares of the perpetrator returning to kill her [his mother]. She witnessed the following interchange between her child and the son of the perpetrator. This child said, “I love my daddy.” Her child responded, “How can you love your daddy, he killed your momma?” The ripple effects of these traumatic deaths clearly affect children, families, friends, and the tight-knit communities themselves.

## Discussion

Results indicated insidious consequences of IPV on indigenous women's lives across multiple dimensions. Women's experiences with IPV had lasting effects across their lives–not only on their mental health–but on their quality of life and personal relationships. Given the tight-knit nature of the indigenous communities and families, the majority of women, not only experienced IPV, they were exposed to it as children and through friends or family members. Thus, even if a woman escaped personally experiencing IPV as an adult, she was likely affected by loved ones’ experiences of IPV. Women who experienced IPV tended to experience especially severe and life-threatening abuse, a finding supported by existing research (Bachman et al., 2008). The consequences of this IPV were concomitantly profound, with women describing PTSD, depression, suicide attempts, sleep disruption, employment problems, and living in constant fear of their perpetrator. These symptoms tended to persist years and even decades beyond the violence.

Consequences of IPV on children were also extensive. Lending support for social learning theory, women expressed the intergenerational nature of violence, where exposure to IPV normalized it as a coping mechanism for conflict resolution resulting in an increased risk for being a victim or perpetrator of IPV (Cannon et al., 2009). Some children modeled behaviors of the perpetrator by participating in berating their mother who was experiencing IPV or striking out aggressively at peers. Boundaries between children of parents experiencing IPV could be diffuse, and children were directly (experiencing child maltreatment and exposure) and indirectly harmed (loss of a parental figure and impaired attachment) (Sturge-Apple et al., 2012). Women who witnessed IPV as a child reported many of the psychological consequences that parallel the consequences of experiencing IPV, including PTSD, bipolar disorder, insomnia, depression, and suicide attempts. Women reported related psychological consequences of their own children who witnessed IPV, including self-harm, suicide attempts, aggressive behaviors, and anger.

Finally, the consequences of IPV on family included what appeared to be secondary trauma and fear that their loved one would be severely hurt or killed. Women who witnessed IPV in their upbringing described family members being victims and perpetrators of IPV, further supporting the intergenerational transmission of violence (Cannon et al., 2009). Whole families were reportedly affected by women being killed in IPV incidents, making the examination of familial and even community effects of IPV pertinent.

### Strengths, limitations, and implications

This culturally sensitive research highlighted the voices of women who were directly and indirectly exposed to violence as children, adults, and family members, using a culturally congruent and rigorous ethnographic method with extensive validity requirements added to its credibility and trustworthiness. Women's recollections brought their lived-experiences of IPV and witnessing IPV to the forefront in a way that is absent in existing research. In addition to psychological consequences, this research added understanding to the consequences on women's subsequent intimate relationships, such as decision making and responsibility, and this information may explain how experiencing IPV places women at risk for subsequent abuse. The profound consequences of experiencing IPV or witnessing parents’ violence provided insight into some of the ways witnessing IPV may affect children's psychological development across the lifespan. Few studies examine the ripple effects of IPV on family, and this investigation documented the extensive consequences on family members. This research has also demonstrated the importance of qualitative research in the study of effects of IPV on women, children, and families by bringing to the fore the complex experiences and consequences of suffering IPV.

Limitations of this research included information being based on self-report from adult women. Despite themes being strikingly consistent across women, the reliability of self-report data can vary. Because some women recalled memories from the past, this information may differ from recollections of those who currently witness or experience IPV. Qualitative results cannot be generalized to other indigenous communities, but many factors may translate to other communities. This is especially the case given the close-knit nature of many indigenous communities. Although there may be variances across indigenous populations, family has been a central theme and may serve as a protective or risk factor, depending on the circumstances (Burnette, Sanders, Butcher, & Rand, 2014). Given the consequences of IPV on children and families within the general population, these results likely transcend tribal borders and are applicable across multiple contexts.

Although this research is specific to indigenous communities in the United States, results likely translate to other tight-knit families across international contexts. These findings highlight the interconnected consequences of IPV across individual and familial levels, and reveal a gap in knowledge about these likely interconnections among the general population. Thus, these qualitative findings can be incorporated into future qualitative and quantitative research to evaluate their relevance across contexts. Distinct factors that emerged within results, such as the consequences of IPV on women's decision making, future relationships, and family processes; these factors can be incorporated into survey-research or in-depth qualitative research to explain their underlying mechanisms related to IPV. Future research on the perspectives of perpetrators, children, families, and professionals would add important information to existing knowledge. Comparing across indigenous communities with more localized examinations of the consequences of IPV on indigenous populations is needed, given contextual variability. Finally, culturally specific interventions incorporating the whole family are needed, given the ramifications of IPV throughout the family and across generations.
